# The influence of constraints on the efficient allocation of resources for HIV prevention

**DOI:** 10.1097/QAD.0000000000002158

**Published:** 2019-02-07

**Authors:** Isaac J. Stopard, Jessica B. McGillen, Katharina Hauck, Timothy B. Hallett

**Affiliations:** Department of Infectious Disease Epidemiology, Imperial College London, London, UK.

**Keywords:** AIDS, allocative efficiency, cost-effectiveness analysis, mathematical modelling, technical efficiency

## Abstract

**Objective:**

To investigate how ‘real-world’ constraints on the allocative and technical efficiency of HIV prevention programmes affect resource allocation and the number of infections averted.

**Design:**

Epidemiological modelling and economic analyses in Benin, South Africa and Tanzania.

**Methods:**

We simulated different HIV prevention programmes, and first determined the most efficient allocation of resources, in which the HIV prevention budget is shared among specific interventions, risk-groups and provinces to maximize the number of infections averted. We then identified the efficient allocation of resources and achievable impact given the following constraints to allocative efficiency: earmarking [provinces with budgets fund pre-exposure prophylaxis (PrEP) for low-risk women first], meeting targets [provinces with budgets fund universal test-and-treat (UTT) first] and minimizing changes in the geographical distribution of funds. We modelled technical inefficiencies as a reduction in the coverage of PrEP or UTT, which were factored into the resource allocation process or took effect following the allocation. Each scenario was investigated over a range of budgets, such that the impact reaches its maximum.

**Results:**

The ‘earmarking’, ‘meeting targets’ and ‘minimizing change’ constraints reduce the potential impact of HIV prevention programmes, but at the higher budgets these constraints have little to no effect (approximately 35 billion US$ in Tanzania). Over-estimating technical efficiency can result in a loss of impact compared to what would be possible if technical efficiencies were known accurately.

**Conclusion:**

Failing to account for constraints on allocative and technical efficiency can result in the overestimation of the health gains possible, and for technical inefficiencies the allocation of an inefficient strategy.

## Abstract 2

Supplemental Digital Content is available in the text

## Introduction

A recent theme in HIV research has been the use of simulation models and cost-effectiveness analyses (CEAs) to inform the ‘socially optimal’ allocation of resources for HIV/AIDS treatment and prevention [1,2]. Models are useful for estimating the impact of existing or planned public health programmes [3], and CEAs are used to identify the set of health interventions that maximizes some social objective, typically aggregate health gains measured in directly comparable natural units, subject to a budget constraint [4]. Ensuring the most efficient (health maximizing) allocation of resources will require allocative efficiency (targeting resources among intervention types, places and population groups to maximize health gains) and technical efficiency (minimizing waste to maximize the gains from resources given their allocation) [5]. Consequently, numerous analyses have demonstrated the reductions in infections or mortality that could be achieved by the most efficient HIV prevention programmes that can be funded with a given budget [2,6–9].

However, these analyses tend to be naïve to the constraints under which health programmes operate. Typically, they assume a single constraint (i.e. a fixed budget), whereas decision makers must consider many additional constraints [10–12]. Common constraints, such as the structure of existing health systems, transition costs associated with altering strategies, weak governance and political constraints (e.g. earmarks imposed by donors or needing to meet a target) may all influence the interventions that are actually implemented [11]. Furthermore, practical supply-side and demand-side constraints, such as deficiencies in supply chains and barriers that impede access to healthcare (e.g. stigma of patients and healthcare providers [13], or patients’ monetary and non-monetary costs of accessing care [10]) can reduce the technical efficiency with which resources are converted to health outcomes. Failure to consider such constraints to allocative and technical efficiency when attempting to identify the most efficient resource allocation may therefore limit the usefulness of CEAs.

Here we theoretically model the HIV epidemic, in Benin, South Africa and Tanzania, given different HIV prevention programmes. The impact of ‘real-world’ constraints on the resource allocation and possible health gains are investigated.

## Methods

### Simulation model design

Building on our previous work [2,6], we used a deterministic compartmental model to represent sexual HIV transmission among adults in the top-level administrative subnational regions (provinces) of Tanzania. The analysis was repeated for Benin and South Africa to investigate generalizability.

Four intervention types could be modelled: behavioural change communication, pre-exposure prophylaxis (PrEP), voluntary medical male circumcision and universal test-and-treat (UTT) services. UTT consists of community outreach and testing services, such that individuals are diagnosed and start antiretroviral therapy (ART), on average, at an earlier stage of disease progression. In all cases, the model is run 2016–2030, assuming HIV prevention scale-up and HIV prevention budgets (henceforth referred to as ‘budget’) begin in 2016.

### Resource allocation under ‘real-world’ constraints

We first conduct an exhaustive search of all intervention combinations to determine the ‘pure efficiency’ solution to the allocation of resources. We only investigate interventions that have HIV prevention as their objective. Therefore, for a given budget, the cost-effective (‘incidence minimizing’) solution is calculated as the distribution of expenditure between interventions and population subgroups that maximizes the number of infections averted relative to a ‘basic treatment’ only scenario, whereby ART is provided to 90% of those with a CD4^+^ cell count of less than 350 cells/μl.

We then identified the cost-effective resource allocations that meet the requirements of additional ‘real world’ constraints (Table 1).

**Table 1:** The modelled constraints on allocative efficiency.

Name	Representation	Assumptions
‘Earmarking’	Multiple criteria apart from efficiency (such as equity and cultural), and priorities of differing stakeholders must be taken into account by decision makers, often in an ad-hoc manner [14]. For example, due to specific criteria donors may attach specific conditions on how funds are spent, thereby limiting the health interventions, for specific diseases or key populations, decision makers can implement [15]	We stipulated that if a province received funding the first intervention funded would be PrEP for heterosexual women (excluding FSWs)
‘Meeting targets’	Externally imposed targets and the political context may influence the allocation process [11]	We stipulated that if a province received any prevention funding then, as a proxy for UNAIDS 90–90–90 target implementation [16], 90% of PLHIV must receive UTT
‘Minimizing change’	Decision makers may have limited capacity to modify existing HIV prevention programmes or redirect funding to different provinces due to the cost of making changes, such as the investments required for new clinics or personnel training [11]	We defined a simple weighted capitation resource allocation between provinces, approximated by the resource allocation that would arise if funds were distributed to maximize HIV prevention but with a single nationwide strategy. Under the ‘minimizing change’ constraint the total budget allocated to a province was the same as that in the previously defined weighted capitation allocation
All constraints on allocative efficiency		The earmarking, meeting targets and minimizing change constraints were applied simultaneously

FSW, female sex worker; PLHIV, people living with HIV; PrEP, preexposure prophylaxis; UTT, universal test-and-treat; UNAIDS, The Joint United Nations Programme on HIV/AIDS.

Conceptually, we assume that technical inefficiencies can act at multiple stages in HIV prevention programmes:

(1) ‘Implementation stage’ – resources have been allocated assuming a certain coverage level is achievable, but if these coverage levels are not achieved we assume resources are wasted.
(2) ‘Allocation and early implementation stage’ – we allow for the possibility that technical inefficiencies are recognized during the allocation and early implementation stage, and the resource allocation can be adjusted in anticipation of lower coverage levels being achievable.

For both these scenarios, we model constraints on technical efficiency as capping achievable UTT coverage at 45% (original assumption is 90% for the efficient allocation) or the coverage of PrEP to 25% for female sex workers (FSW) and MSM, and 12.5% for other men and women (original assumption is 50 and 25%, respectively).

It is important to note all scenarios are subject to three constraints. First, the budget is fixed. Second, we assume preset population coverage levels for each intervention, which stipulates that interventions are not scalable and either implemented at a specific coverage level or not at all. This is an assumption of equity in intervention access and reduces modelling complexity. Third, ‘basic treatment’ is always provided and does not impact the prevention budget.

For each scenario, we investigated the allocation of resources for a hypothetical range of budgets such that the complete impact range is investigated [0 billion (Bn) US$ to approximately 35 Bn US$ for Tanzania]. As a benchmark, the total budget for Tanzania was approximately 9.5 Bn US$, assuming The United States President's Emergency Plan For AIDS Relief (PEPFAR) and Global Fund yearly contributions in 2015 and 2014, respectively [17] remain constant. Intervention costs and maximum achievable population coverages were consistent with our previous work [2], with the exception of UTT coverage, which in accordance with the Joint United Nations Programme on HIV/AIDS (UNAIDS) 90–90–90 targets [16] was set at 90%. Unit costs were assumed to be constant across populations and provinces.

## Results

**Fig. 1:**
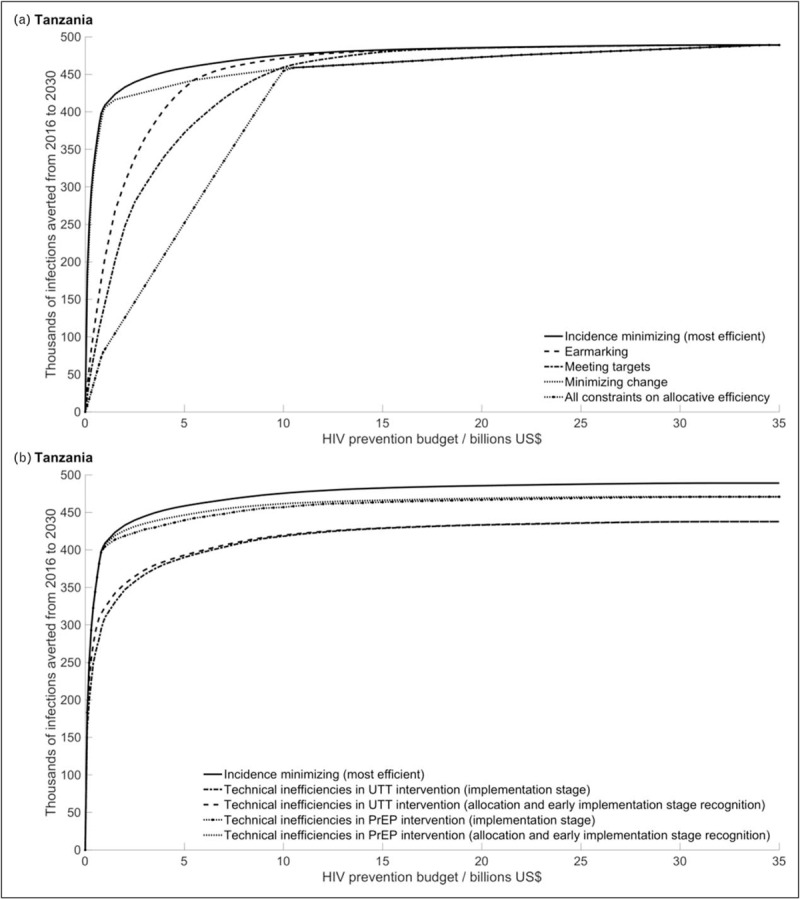
Differences in the HIV prevention efficiency of the ‘incidence minimizing’ and constrained scenarios.

In Tanzania, the ‘incidence minimizing’ allocation could avert approximately 183 000 infections, for a budget of 0.1 Bn US$, to a maximum of approximately 489 000 infections, for approximately 32 Bn US$, between 2016 and 2030 (Fig. 1). At lower expenditures, budget increments allow the most cost-effective interventions to be scaled up among more populations, giving large returns in HIV prevention.

Simulation of the number of infections averted (relative to a ‘basic treatment’ scenario) over the period 2016–2030, at each HIV prevention budget for the most efficient (incidence minimizing) and scenarios with constraints to allocative efficiency (a) or technical efficiency (b). The previously described model was simulated with the calibrations of McGillen *et al.*[2].

### Constraints on allocative efficiency

Imposing constraints on allocative efficiency reduces the number of infections averted relative to the ‘incidence minimizing’ allocation, up until a given budget at which there is no difference between the scenarios (Fig. 1a).

The ‘earmarking’ and ‘meeting targets’ constraints have large impacts at lower budgets. For example, given a budget of 0.1 Bn US$ these constraints reduce the number of infections averted by 82 and 89%, respectively. Both these constraints require the inclusion of arbitrary interventions within the resource allocation; at lower budgets the impacts of these constraints therefore depend on the alignment (inclusion of the specific interventions) with the ‘incidence minimizing’ resource allocation. The ‘meeting targets’ constraint, which requires UTT for everyone, is greater than the ‘earmarking’ constraint, which only requires PrEP for heterosexual women (excluding FSWs). Given a budget of 32 Bn US$ (the budget that results in the maximum number of infections averted in the ‘incidence minimizing’ scenario) the ‘earmarking’ and ‘meeting targets’ have negligible impact and the number of infections averted between these three scenarios converge.

The ‘minimizing change’ constraint has less of an impact at lower budgets (11% reduction in the number of infections averted relative to the ‘incidence minimizing’ scenario), but has a greater impact at higher budgets than the other modelled constraints to allocative efficiency (0.5% at 32 Bn US$) and does not converge with the ‘incidence minimizing’ resource allocation until approximately 34–34.5 Bn US$.

When all these constraints on allocative efficiency are applied together, the reduction in infections averted relative to the ‘incidence minimizing’ allocation would be between 96% (at 0.1 Bn US$) and 0.5% (at 32 Bn US$).

### Constraints on technical efficiency

Technical inefficiencies also reduce the health impacts (Fig. 1b). If considered during the allocation process and decisions are adapted (Fig. 1b), then the effect of reduced intervention coverage depends on whether that intervention is selected during the CEA. For example, at a lower budget (0.1 Bn US$), UTT technical inefficiency causes a 6% decrease in infections averted relative to the ‘incidence minimizing’ scenario. But, PrEP technical inefficiency has no impact, as it is not implemented at this budget. At a higher budget (32 Bn US$) the lower UTT and PrEP coverages reduce the number of infections relative to the ‘incidence minimizing’ allocation by 11 and 4%, respectively.

If technical inefficiencies are not considered during the allocation but take effect when the interventions are applied (Fig. 1b) the reduction in health impact at lower budgets is even greater (12 and 0.2% at 0.1 Bn US$ for a 50% reduction in UTT and PrEP coverage, respectively). This is because funds are allocated to an intervention with the expectation that a certain health gain will be achieved, but if this gain is not achievable then it could have been better to allocate the funds elsewhere.

### Generalizability

We ran the analyses for Benin and South Africa, finding the results are qualitatively reproduced (refer to Figs. S1–S4 in the Supplementary information). The ‘meeting targets’ constraint has greater impact in South Africa and the ‘minimizing change’ constraint is weaker in both settings, suggesting the predicted current distribution of funds between provinces is close to the most efficient distribution. In Benin, considering technical inefficiencies during the allocation process has little impact.

## Discussion

With a focus on reducing HIV incidence in Tanzania, our theoretical model demonstrates that failing to consider certain real-world constraints under which decision makers operate can lead to a situation whereby models showing the most efficient resource allocation for HIV prevention: over-estimates possible impact, and misdirects resources, potentially reducing actual health gain.

Previous studies have recognized the need [10,11], outlined a conceptual framework [12] or adjustments to cost-effectiveness decision-making [18] to incorporate ‘real-world’ supply-side or demand-side constraints in modelling the efficient allocation of resources. Hontelez *et al.*[19] modelled the removal of limited health system capacity to provide ART and/or ART demand, finding removal of these constraints increased the health gains. However, a review of cost-effective analyses of the scale-up of ART, in sub-Saharan Africa, found that of 34 studies, few included at least one supply-side (4) or demand-side (11) constraint [10].

The constraints to allocative efficiency we have modelled are, of necessity, arbitrary but rooted in reality. For instance, a recent assessment of Ghanaian and Ugandan national policy indicated the transfer of the UNAIDS 90–90–90 targets into programmatic activities [20], and PEPFAR funding comes to countries with earmarks for certain initiatives [21]. Furthermore, in South Africa, from 1996 to 2007 provinces with greater capacity to spend funds (i.e. existing health infrastructure) received more funds [22], thus indicating the potential for existing health systems to impact geographical distribution of funds. These examples therefore indicate the relevance of our ‘meeting targets’, ‘earmarking’ and ‘minimizing change’ constraints, respectively.

Estimates of technical efficiency are often derived from experimental evidence of highly monitored facilities, the findings from which may not be consistent among health facilities [5]. Social and economic structural barriers, such as stigma and patient costs of accessing healthcare, can prevent the uptake and adherence to HIV interventions and treatment. These are not uniform and are hard to characterize fully [23–28] although, more recently, research into the accurate estimation of unit costs and technical efficiencies of different HIV intervention types across geographical regions and risk groups [29], and the impact of economies of scale [30] have been carried out. Furthermore, for some purposes, deliberately ambitious targets and high expectations of efficiency may be selected.

We have not accounted for other potential effects of the modelled constraints. For example, the procurement policies of global large-scale antiretroviral drug purchasers, such as PEPFAR, and the advice of international organizations can influence market prices and consequently cost-effectiveness [31,32]. Thus, indicating the potential for interactions between cost-effectiveness and the actions of large-scale funders. Furthermore, we do not conduct a cost-utility analysis and consider the additional health benefits that can occur. For example, diagnosis and access to ART for people living with HIV, for example UTT, has been demonstrated to improve both quality of life and life expectancy [33,34].

In conclusion, our results demonstrate that failing to account for ‘real-world’ constraints can over-estimate the health gains that are achievable and incomplete consideration of technical inefficiencies can result in the allocation of an inefficient strategy. These findings therefore indicate that to designate the most cost-effective intervention strategy, modellers must incorporate the range of relevant constraints.

## Acknowledgements

I.J.S. thanks S. Bajaj for proof-reading early drafts.

Authors’ contributions: I.J.S., J.B.M., K.H. and T.B.H. conceived the study. I.J.S., J.B.M. and T.B.H. developed the methods. I.J.S. and J.B.M. conducted the analyses and programmed the constraints. I.J.S., K.H. and T.B.H. wrote the report.

Funding for this study was provided by the Bill & Melinda Gates Foundation through a grant (OPP1084364) to the HIV Modelling Consortium at Imperial College London. We acknowledge the joint MRC Centre for Global Infectious Disease Analysis funding from the UK Medical Research Council and Department for International Development (grant reference: MR/R015600/1).

Submission: AIDS (Concise Communication).

### Conflicts of interest

There are no conflicts of interest.

## Supplementary Material

Supplemental Digital Content
