# Supplementary material for: The influence of constraints on the efficient allocation of resources for HIV prevention
Source: AIDS. 2019 Feb 7;33(7):1241–6. doi: 10.1097/QAD.0000000000002158 (PMC6511422; doi:10.1097/QAD.0000000000002158)
Supplement: Supplemental Digital Content [file aids-33-1241-s001.doc]

Supplementary Information


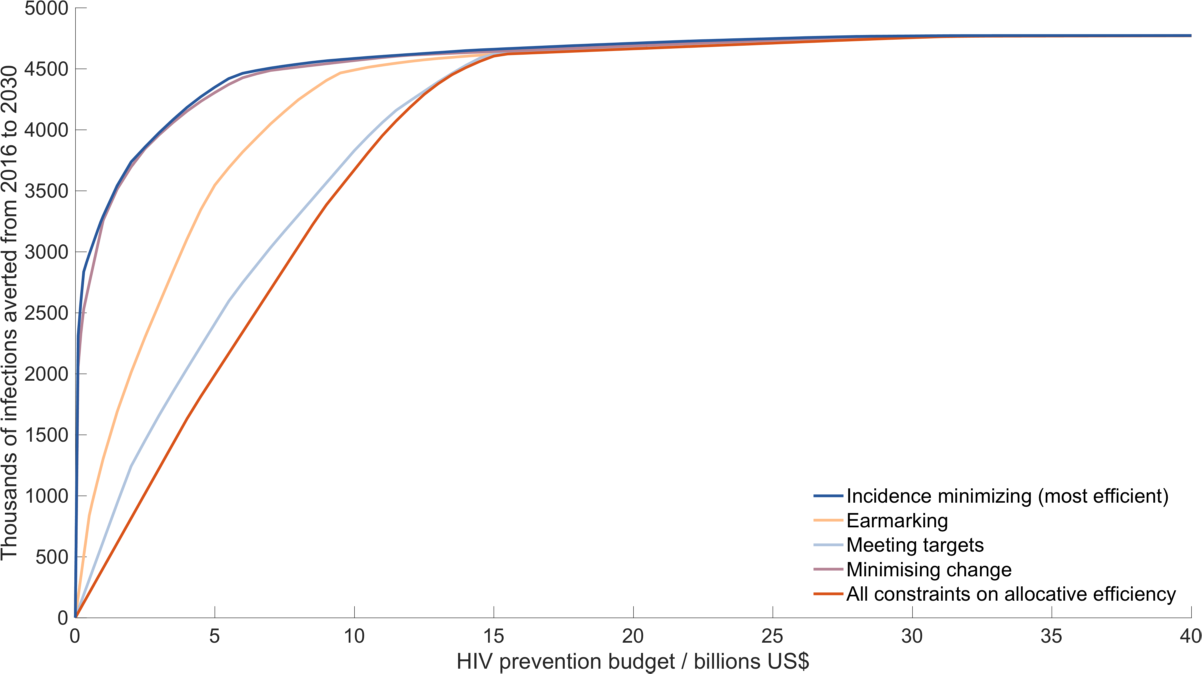


**Figure S1. Differences in HIV prevention programme outcomes of the ‘incidence minimizing’ and scenarios with constraints to allocative efficiency, in South Africa.** The y-axis shows the simulated number of infections averted (relative to a ‘basic treatment’ scenario), over the period 2016-2030, at each HIV prevention budget. The following constraints to allocative efficiency are modelled: earmarking (PrEP for heterosexual women [excluding FSW] is funded first), meeting targets (90% of PLHIV must receive UTT prior to funding alternative interventions) and minimizing change (the distribution of funds between provinces is maintained at the level defined by a weighted capitation allocation). A deterministic compartmental transmission model with the calibrations of McGillen et al. ^[1]^ was used to represent sexual HIV transmission.


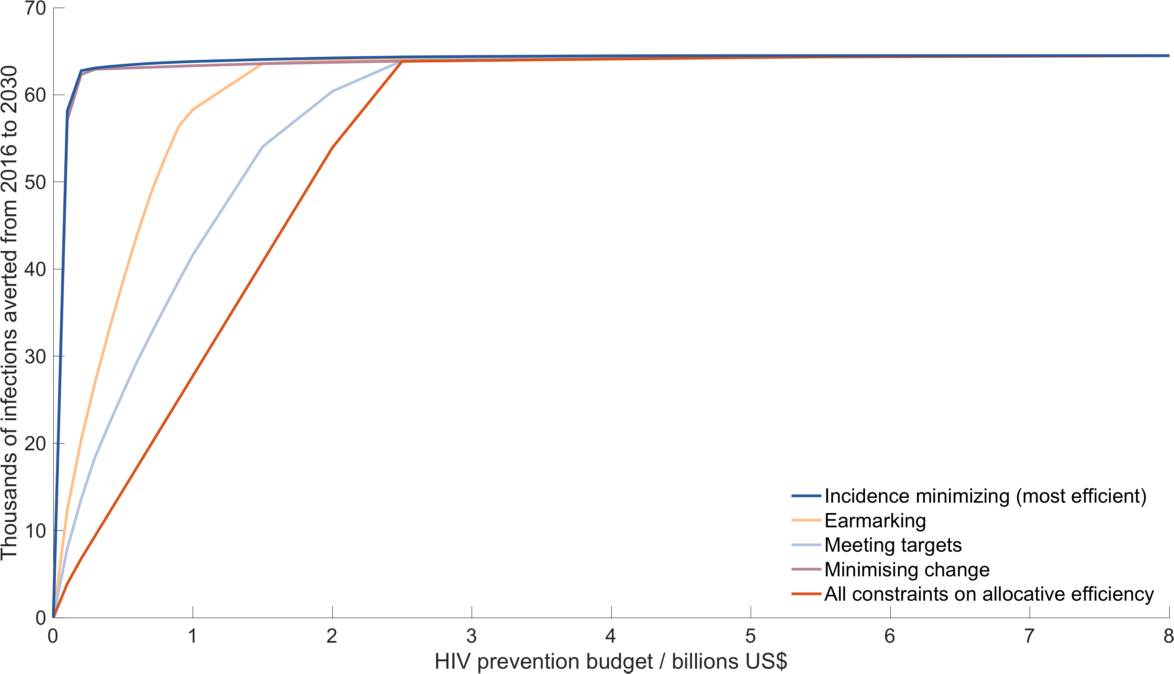


**Figure S2. Differences in HIV prevention programme outcomes of the ‘incidence minimizing’ and scenarios with constraints to allocative efficiency, in Benin.** The y-axis shows the simulated number of infections averted (relative to a ‘basic treatment’ scenario), over the period 2016-2030, at each HIV prevention budget. The following constraints to allocative efficiency are modelled: earmarking (PrEP for heterosexual women [excluding FSW] is funded first), meeting targets (90% of PLHIV must receive UTT prior to funding alternative interventions) and minimizing change (the distribution of funds between provinces is maintained at the level defined by a weighted capitation allocation). A deterministic compartmental transmission model with the calibrations of McGillen et al. ^[1]^ was used to represent sexual HIV transmission.


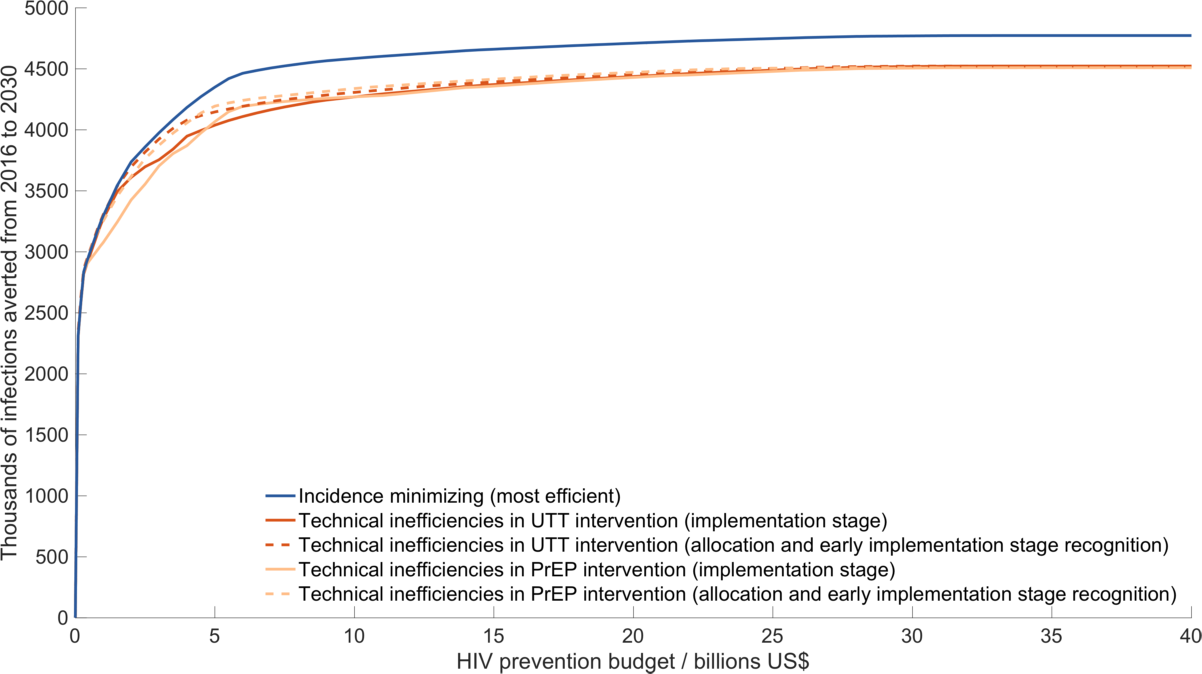


**Figure S3. Differences in HIV prevention programme outcomes of the ‘incidence minimizing’ and scenarios with constraints to technical efficiency, in South Africa.** The y-axis shows the simulated number of infections averted (relative to a ‘basic treatment’ scenario), over the period 2016-2030, at each HIV prevention budget. Technical inefficiencies are modelled by reducing PrEP or UTT coverage by half relative to the incidence minimizing scenario, these reductions can be recognised during the allocation process and factored into the resource allocation process (allocation and early implementation stage recognition) or not (implementation stage). A deterministic compartmental transmission model with the calibrations of McGillen et al. ^[1]^ was used to represent sexual HIV transmission.


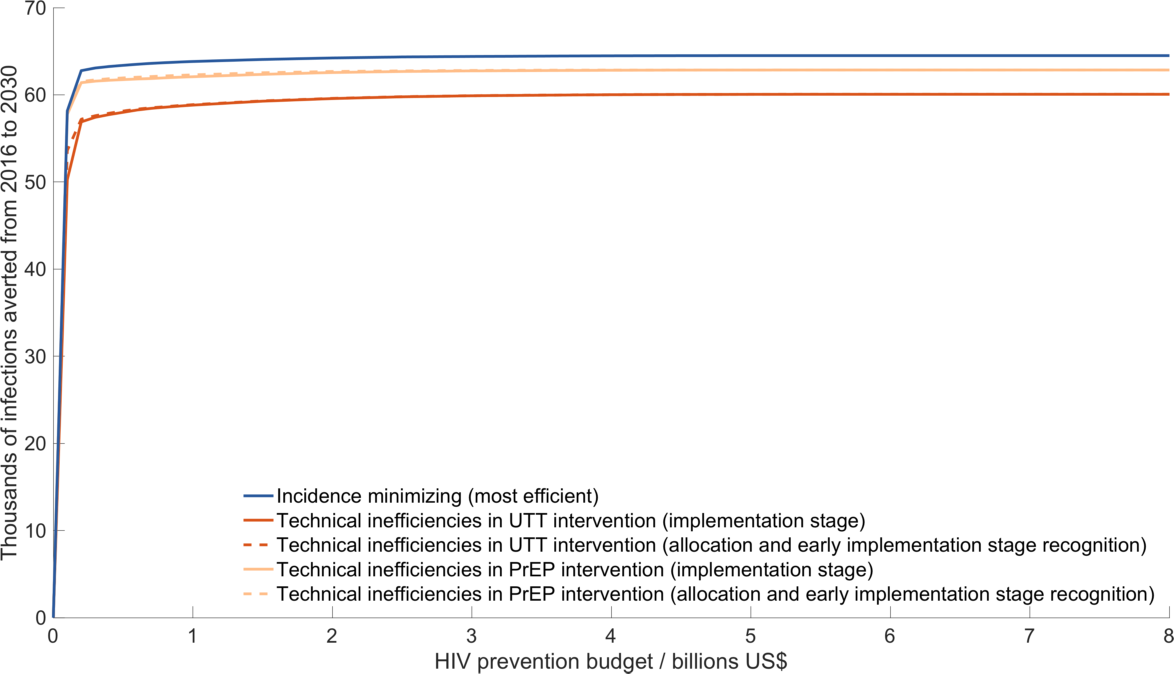


**Figure S4. Differences in HIV prevention programme outcomes of the ‘incidence minimizing’ and scenarios with constraints to technical efficiency, in Benin.** The y-axis shows the simulated number of infections averted (relative to a ‘basic treatment’ scenario), over the period 2016-2030, at each HIV prevention budget. Technical inefficiencies are modelled by reducing PrEP or UTT coverage by half relative to the incidence minimizing scenario, these reductions can be recognised during the allocation process and factored into the resource allocation process (allocation and early implementation stage recognition) or not (implementation stage). A deterministic compartmental transmission model with the calibrations of McGillen et al. ^[1]^ was used to represent sexual HIV transmission.

References

1. McGillen JB, Anderson SJ, Dybul MR, Hallett TB**. Optimum resource allocation to reduce HIV incidence across sub-Saharan Africa: a mathematical modelling stu**dy*. Lancet HI*V 2016; 3(9):E441-E448.
